# Oxidative Stress and Metabolic Syndrome: Cause or Consequence of Alzheimer's Disease?

**DOI:** 10.1155/2014/497802

**Published:** 2014-01-20

**Authors:** Diana Luque-Contreras, Karla Carvajal, Danira Toral-Rios, Diana Franco-Bocanegra, Victoria Campos-Peña

**Affiliations:** ^1^Facultad de Ciencias Químicas, Universidad Autónoma de Coahuila, Boulevard V. Carranza S/N, Colonia República Oriente, Saltillo, COAH, Mexico; ^2^Laboratorio de Nutrición Experimental, Instituto Nacional de Pediatría, Insurgentes Sur 3700 letra C, Coyoacán, 04530 Mexico City, Mexico; ^3^Departamento de Fisiología Biofísica y Neurociencias, Centro de Investigación y de Estudios Avanzados del Instituto Politécnico Nacional, Instituto Politécnico Nacional, 2508, 07360 Mexico City, Mexico; ^4^Universidad Nacional Autónoma de México, Avenida Insurgentes Sur 3000, Coyoacán, 04510 Mexico City, Mexico; ^5^Laboratorio Experimental de Enfermedades Neurodegenerativas, Instituto Nacional de Neurología y Neurocirugía Manuel Velasco Suárez, Insurgentes Sur 3877, 14269 Mexico City, Mexico

## Abstract

Alzheimer's disease (AD) is a major neurodegenerative disease affecting the elderly. Clinically, it is characterized by a progressive loss of memory and cognitive function. Neuropathologically, it is characterized by the presence of extracellular *β*-amyloid (A*β*) deposited as neuritic plaques (NP) and neurofibrillary tangles (NFT) made of abnormal and hyperphosphorylated tau protein. These lesions are capable of generating the neuronal damage that leads to cell death and cognitive failure through the generation of reactive oxygen species (ROS). Evidence indicates the critical role of A*β* metabolism in prompting the oxidative stress observed in AD patients. However, it has also been proposed that oxidative damage precedes the onset of clinical and pathological AD symptoms, including amyloid-*β* deposition, neurofibrillary tangle formation, vascular malfunction, metabolic syndrome, and cognitive decline. This paper provides a brief description of the three main proteins associated with the development of the disease (A*β*, tau, and ApoE) and describes their role in the generation of oxidative stress. Finally, we describe the mitochondrial alterations that are generated by A*β* and examine the relationship of vascular damage which is a potential prognostic tool of metabolic syndrome. In addition, new therapeutic approaches targeting ROS sources and metabolic support were reported.

## 1. Introduction

It has been speculated that the free radicals produced during oxidative stress are pathologically important in AD and other neurodegenerative diseases. Oxidative stress can be defined as an imbalance between ROS production and/or their elimination. That oxidative stress implicated in the etiology of AD is possibly due to changes in the redox status that occur in AD brains [1]. In recent years, it has been proposed that not only oxidative stress is a significant early event in the development of the disease, but also it plays an important role in modulating signaling pathways leading to cell death. Recent evidence has suggested that the presence of *β*-amyloid is crucial in the development of the pathology. A*β* results from the sequential proteolysis of the amyloid precursor protein (A*β*PP) by *β*-secretase (BACE1) and *γ*-secretase, a multiprotein complex. While, under physiological conditions, A*β* appears to be unfolded, in pathological conditions, it is proposed that it increases the production of amyloid or its ability to aggregate [2, 3]. A*β* toxicity is dependent on A*β*'s conformational state, peptide length, and concentration [4–8]. A*β* deposition in the brain occurs not only in the parenchyma but also in the vessel walls, causing cerebral amyloid angiopathy (CAA), which is another pathological phenomenon commonly found in the AD brain. Regarding the pathogenic role of CAA in AD, it has been increasingly recognized that vascular pathology constitutes a risk factor for AD. These vascular changes are important as predictors for the development of MS. Although the exact mechanisms underlying the connection between MS and AD remain uncertain, it is known that, together, amyloid deposition, vascular damage, impairment of energy metabolism, and insulin resistance are physiological conditions that favor the development of AD.

## 2. Amyloid-*β*

According to the amyloid hypothesis, A*β* peptide accumulation in patients' brain is the key event leading to the development of the pathology. A*β* peptides range from 39 to 42 amino acid residues and have a molecular weight of 4 kD, with the most abundant being A*β*40 peptide, which generates between 80 and 90% of the total A*β* produced. As A*β*42 is only produced by approximately 10% of people, this is more hydrophobic than A*β*40 and is therefore found in greater proportion in the NP of AD patients. In pathological conditions, such as AD, the ratio between changes in A*β*40 and A*β*42 is found to be about 50% each. *In vitro* studies have demonstrated that the incubation of the peptide with cells in culture induces a neurotoxic effect characterized by oxidative stress, apoptosis, and damage to membrane and cytoplasmic proteins, mitochondrial DNA, and lipids [9, 10]. A*β* peptide induces the production of different oxidative adducts that could promote synaptic and mitochondrial dysfunction and cellular apoptosis [9]. Within the A*β* sequence, it has been suggested that methionine 35 plays an important role in promoting oxidative activity. When this amino acid is substituted for another, the oxidative capacity of A*β* is greatly diminished [10–12].

It has been proposed that the amyloid oligomers can insert themselves into the lipid bilayer and cause lipid peroxidation and, consequently, oxidative damage to proteins and other biomolecules [13]. As a result of alteration in the membrane, there is a massive influx of Ca^2+^, which alters the homeostasis of Ca^2+^ causing mitochondrial dysfunction, synapse loss, and, finally, neuronal death. In this regard, it has been widely described that oligomeric A*β* is considered as the most highly toxic form of the protein. It is also known that these oligomeric forms may be produced through several routes, both in the extracellular space and the interior of the cell organelles, such as the endoplasmic reticulum and mitochondria.

## 3. Tau

Tau is a major microtubule-associated protein, which promotes microtubule (MTs) assembly and stability, and becomes essential for the axonal transport of the neuron [14]. Adult human brains have 6 isoforms [15] and contain two domains: the projection domain located in the N-terminal and the microtubule-binding domain (MTBD) in the C-terminal, which is comprised by the presence of three (3R) to four repeats (4R), which performs the interaction with MTs [16, 17]. Although it is a highly soluble and heat-stable protein, NMR studies showed the presence of 8–10 residues with *β*-structure, which, located between the second and third repeat, confer a propensity for aggregation [18]. In AD, the tau protein undergoes oligomerization and forms paired helical filaments (PHFs), which then leads to the development of NFT [19]. Several studies in the literature indicate that prefibrillar stages of tau could be mainly linked with the neurotoxic effects observed in the neurons [20, 21]. It has been reported that tau induces mitochondrial dysfunction, leading to severe energy dysfunction and the generation of ROS and nitrogen species (RNS) [22], which could also disturb the integrity of biological membranes and induce synaptic failure [23]. Although it is not known exactly what generates tau aggregation, *in vivo* and *in vitro* studies have shown that this phenomenon may be triggered by altered posttranslational modifications (phosphorylation, truncation, nitration, ubiquitination, oxidation, and glycation) [24]. Furthermore, the expression of tau truncated at Asp-421-induced mitochondrial fragmentation and elevated oxidative stress levels in comparison with cells expressing full-length tau [25].

*In vitro* and *in vivo* studies have reported that fibrils and A*β* oligomers also induce the conversion of monomeric human tau into *β*-sheet rich toxic tau oligomers [26, 27]. Other cofactors in tau aggregation are metal ions, such as Fe^3+^ and Al^3+^ that coexist in NFT [28] and carry out redox activity, which is catalytic for the generation of free radicals and represents a potential risk of oxidative damage in AD pathology [29]. Oxidative stress could be an early inducer of tau aggregation, due to the fact that the 3xTg-AD mouse presented decreased antioxidant levels and increased levels of lipid peroxidation, before the appearance of NFT [30]. Moreover, chronic oxidative stress and the subsequent formation of 4-hydroxynonenal (HNE) may contribute to tau hyperphosphorylation and induce conformational changes that could lead to the assembly of tau and the formation of NFT [31, 32]. Meanwhile, the peroxynitrite has been shown to be involved in tau nitration, which inhibits the assembly of tau with the MTs and subsequently promotes their oligomerization [33].

In summary, oxidative stress is associated with AD pathology that induces conformational changes or posttranslational modifications that favor tau aggregation. Although it has been suggested that the aggregation is a form of protection against oxidative damage, it also has been proposed that these aggregates promote the generation of ROS, which indicate feedback that is not favorable for cell viability and, therefore, requires further study.

## 4. Apolipoprotein E

The apolipoprotein E (ApoE) is an important protein for maintaining the structural and functional integrity of synapses and membranes [34]. There are three isoforms: ApoE2 (Cys^112^, Cys^158^), ApoE3 (Cys^112^, Arg^158^), and ApoE4 (Arg^112^, Arg^158^) [35]. ApoE4 allele carriers were found to have a higher risk of both AD and also an early onset of the disease in a dose-dependent manner [36]. Different *in vitro* and *in vivo* studies have shown that interaction between apoE and soluble A*β* leads to fibrillization [37, 38]. Interestingly, apoE4 is most efficient in binding intermediate aggregates of A*β*, even more than apoE2 [39]. In addition, apoE4 may increase the intracellular recycling of APP, which could increase A*β* production [40].

On the other hand, studies in the ApoE-deficient mice (ApoE^−/−^) have reported a reduction in the extent of oxidative stress, suggesting a protective role for ApoE [41, 42]. In this way, the transfection of different ApoE isoforms into B12 cells cultures and the posterior exposure to H_2_O_2_ or amyloid (A*β*_25–35_) shows that apoE2 has better antioxidant effects and offers more protection against A*β* toxicity than apoE3 and apoE4 [43]. This isoform-dependent protective activity has been reported in several models, suggesting that apoE4 might represent a risk for the potential loss of the antioxidant system in AD [44, 45]. Due to the strong association between apoE in oxidative damage and AD, efforts have been made to find pharmacological strategies aimed at the use of antioxidants in carriers of *ε*4 risk allele. In this sense, Ginkgo biloba extract (EGb 761) and the neurosteroid dehydroepiandrosterone (DHEA) were able to prevent the *in vitro* oxidant-induction of lipid peroxidation in tissues from AD cases with the *ε*3/3 and *ε*3/4 genotypes [46]. Furthermore, the use of N-acetyl cysteine as a dietary supplement with ApoE^−/−^ mice previously deprived of folate restored glutathione synthase and glutathione levels and alleviated oxidative damage and cognitive decline [47]. The use of vitamin E is controversial because it has been shown to protect against oxidative insults in cell cultures, while no differences in oxidative brain status were observed in mice expressing the ApoE isoforms [48].

So far, it is known that, in the brain, ApoE has a beneficial effect by maintaining lipid homeostasis and contributing to the redox balance. However, in AD, ApoE may contribute to oxidative damage in an isoform-dependent manner, being the ApoE4 isoform that causes more damage.

## 5. Mitochondrial Dysfunction

In every eukaryotic cell, mitochondria are the organelles responsible for providing the necessary energy for metabolic cell processes under aerobic conditions [49]. In neurons, mitochondria have particular importance due to their high aerobic metabolic rates and complex morphology and their role as the provider of adenosine triphosphate (ATP) as a source of energy for neurotransmitter release and recycling. Mitochondrial function in neurons is so crucial in supporting the synaptic machinery, that it is considered a factor limiting synaptogenesis and neuronal plasticity. Indeed, there is a positive correlation between neurite mitochondrial density and the number of dendritic spines [50, 51].

**Figure 1:**
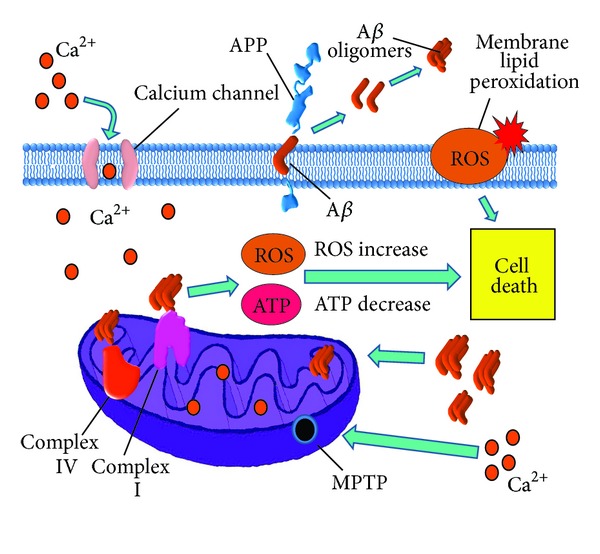
Mitochondrial damage in Alzheimer's disease. Amyloid-*β* (A*β*) overproduction damages mitochondria causing dysfunction of mitochondrial complexes I and IV, which result in reactive oxygen species (ROS) overproduction and adenosine triphosphate (ATP) depletion. In neurons, ATP depletion may lead to neurotransmission dysfunction and altered axonal transport, thus provoking mitochondrial dynamics abnormalities. ATP depletion also causes dysfunction of the ATP-dependent ion channels, leading to altered ion balance in the cytosol. ROS increase in turn leads to mitochondrial permeability transition pore (MPTP) aperture, which increases mitochondrial damage by allowing calcium entrance into the mitochondrial matrix, worsening the electron transport chain and oxidative phosphorylation disruption. ROS overproduction also causes membrane damage due to lipid peroxidation and triggering cell death mechanisms (apoptosis).

Mitochondrial dysfunction has been observed as characteristic of many neurodegenerative diseases, even before other distinctive disease features and symptoms appear. One of the neurodegenerative diseases in which mitochondrial dysfunction occurs is AD. Mitochondrial defects damage the cell in two main ways: (1) by significantly increasing the production and releasing a variety of ROS which, in turn, cause cell damage and eventual death, and (2) by causing energy depletion due to the disruption of oxidative phosphorylation (Figure 1).

Recent studies show that A*β* may be responsible for neuronal death and synapse loss due to the adverse effects it has on mitochondrial structure and function. It has been shown in AD models that overexpressing human APP (hAPP), leads to A*β* accumulation in neuronal mitochondria, which in turn affects mitochondrial function, as described below [52].

### 5.1. A*β*-Induced Electron Transport Chain Dysfunction

One of the ways in which A*β* damages mitochondria is by decreasing the activity of electron transport chain enzymes. Du et al. [52] found that, in the synaptic mitochondria of hAPP mice, both complex IV (cytochrome c oxidase) activity and respiratory control ratio decreased, while oxidative stress (measured as 4-hydroxynonenal and hydrogen peroxide levels) increased in comparison with wild-type (WT) mice. A variety of studies have demonstrated that complex IV dysfunction is able to increase ROS generation. Recent studies have demonstrated that A*β* is able to decrease the activity of complex IV, by binding directly with subunit 1 of the enzyme cytochrome c oxidase. The interaction between A*β*_1–42_ and subunit 1 of the cytochrome c oxidase explains the decrease in the activity of the complex IV enzyme and also, therefore, the metabolic alterations found in the disease. Coimmunoprecipitation assays carried out for A*β*_1–42_ with this subunit confirmed the binding.

It has been also reported by Bobba et al. [53] that, in a primary rat cortical cell culture, treatment with A*β* caused a deficiency of both complex I (NADH dehydrogenase) and complex IV (cytochrome c oxidase). Complex I is one of the main ROS generation sites in mitochondria under normal physiological conditions, and changes in complex I function could be responsible for an increase in ROS production. In this study, it was observed that in cultured neurons treated with A*β* ROS production and lipid peroxidation increased 5-fold when compared with untreated controls.

The electron transport chain dysfunction observed in transgenic AD mice causes energy alterations that can be responsible for synapse loss in two ways. On one hand, it may disrupt neurotransmission due to insufficient ATP supply [54]. On the other hand, because of the ATP deficit, ATP-dependent enzymes become dysfunctional, eventually leading to a collapse of, among others, cellular Na^+^ and Ca^2+^ homeostasis, which are necessary to maintain the right membrane potential in order that synapse can take place [55].

### 5.2. Opening of the Mitochondrial Permeability Transition Pore

Elevated ROS generation induced by A*β* can further damage mitochondria by stimulating the aperture of the mitochondrial permeability transition pore (MPTP). MPTP is a protein complex that forms an unselective channel that passes through both inner and outer mitochondrial membranes. In normal conditions, MPTP has low permeability, but, in some pathological conditions, its permeability dramatically increases, leading to intracellular calcium overload and oxidative stress.

A*β* is thought to stimulate MPTP aperture by increasing intracellular Ca^2+^ through a reduction in Ca^2+^-ATPase activity. In addition, intracellular Ca^2+^ alters the lipid organization of the inner mitochondrial membrane by interacting with cardiolipin, the major phospholipid of this membrane. These alterations may affect the respiratory electron transport chain function, thus generating oxidative stress and inducing the opening of the MPTP [56].

It has been demonstrated in hAPP mice that protein expression of some components of the MPT, such as cyclophilin D (CypD), voltage-dependent anion channel (VDAC), and adenine nucleotide translocator (ANT), is elevated. This indicates that overexpression of APP, and the consequent amyloid overload, may lead to opening of the MPTP, thus disrupting oxidative phosphorylation and ATP production, all of which leads to synaptic loss and eventually cell death [9, 52].

### 5.3. Alterations in Mitochondrial Dynamics

There is evidence to show that A*β* can also have an effect on changing mitochondrial size and dynamics, and it is thought that oxidative stress also has a role in these abnormalities. Calkins et al. [54] found that, in hAPP mice neurons scanned by a transmission electron microscope, mitochondria were on average significantly smaller than mitochondria from WT controls. Wang et al. [57] showed that the expression of proteins involved in sizing and recycling mitochondria, such as dynamin-related protein 1 (Drp1) and fusion proteins, including optic atrophy protein 1 (OPA1), mitofusin 1 (Mfn1), and mitofusin 2 (Mfn2), is reduced, while for fission protein 1 (Fis1), gene expression is increased in AD postmortem hippocampal tissues. These results coincide with those of Manczak et al. [58], who found reduced OPA1, Mfn1, and Mfn2 gene expression and increased Drp1 and Fis1 expression in postmortem AD human brain tissues. All of this suggests enhanced mitochondrial fission conditions leading to a general decrease in mitochondrial size and subsequently affecting cell energy metabolism [59].

A*β* also has an effect in disturbing the mitochondrial dynamics that regulate axonal transport. Du et al. observed that axonal mitochondrial density and anterograde mitochondrial transport are reduced in hippocampal mice cell cultures treated with A*β*, compared to untreated controls [52]. These alterations coincide with previously reported mitochondrial changes in ROS-treated cell cultures [60], thus providing evidence to say that they are likely due to the oxidative stress generated by the aforementioned dysfunctions in the electron transport chain.

### 5.4. Mitochondrial Creatine Kinase

Creatine kinase (CK) is an enzyme that phosphorylates creatine by transferring a phosphate form ATP to build up phosphocreatine. It has four isoforms expressed exclusively in tissues or cells that demand high energy levels, such as those found in the muscle and brain. In the brain, cytosolic CK exists as a homodimer known as brain-type creatine kinase (CK-BB). Mitochondria express both the dimeric and octameric isoenzymes (uMtCK) found in the intermembrane space. Thus, the CK circuit in the brain contributes to the management of the energy supply for neuron functions. Loss of uMtCK has been associated with anomalous hippocampal mossy fiber connections, delayed seizure development, reduced open-field habituation, and slower spatial learning [61, 62]. CK function may be altered in AD, leading to deficits in the maintenance of optimal energy levels and altered energy supply within glia, neurons, and synapses [63].

All of these findings show that mitochondrial dysfunction is an important piece in the puzzle of AD pathology and accounts for a significant proportion of A*β*-induced oxidative stress, which itself, in turn, contributes to neuronal death. Insights into mitochondrial alterations in AD could highlight novel therapeutic targets for AD management and treatment.

## 6. Vascular Endothelium: Oxidative Stress Damage

Recently, alterations in cerebrovascular regulation related to vascular oxidative stress have been implicated in the mechanisms of the early stages of AD [64–66]. There is a growing body of evidence that points to the endothelium as an important culprit implicated in neurodegenerative disorders by oxidative stress damage. It is known that the vascular endothelium, which regulates the passage of macromolecules and circulation of cells from blood to tissue, is a major target for oxidative stress, playing a critical role in the pathophysiology of vascular diseases. Since the vascular endothelium, neurons, and glia are all able to synthesize, store, and release ROS and vascular active substances in response to certain stimuli, their contribution to the AD pathology could be significant [67].

The vascular endothelium is uniquely positioned at the interface between the blood vessel wall and blood flow, where it exerts multiple functions, including the modulation of the vessel tone [68]. Because of its strategic location, the endothelium likely serves as the primary intermediary of mechanotransduction, initiating vascular processes in response to changes in blood flow. When an imbalance between endothelial factors occurs (such as in the case of ROS elevation), the endothelium becomes dysfunctional. This is evidenced by losing the ability to respond to different vascular stimulus such as vasodilation.

There is now a wealth of evidence suggesting that oxidative stress is a major cause of endothelial dysfunction in the cerebral circulation. Genetic and pharmacological interventions to inhibit the major source of reactive oxygen species, nicotinamide adenine dinucleotide phosphate (NADPH) oxidase, are neuroprotective in experimental cerebral ischemia. Also, recent studies have demonstrated that inhibition of NADPH oxidase activity can mitigate cognitive impairment in rodent models of cerebral hypoperfusion [69]. Studies realized by Sochocka et al. showed that the vascular endothelium and chronic hypoperfusion may play an important role in the pathobiology of AD. Hypoperfusion appears to induce oxidative stress, and over time this damage could initiate mitochondrial failure, which is known as a primary factor in AD [70]. Recent evidence indicates that chronic injury stimulus induces the hypoperfusion seen in vulnerable brain regions. This reduced regional cerebral blood flow (CBF) leads to energy failure within the vascular endothelium and associated brain parenchyma, manifested by mitochondrial ultrastructure damage and by the overproduction of mitochondrial DNA deletions [71]. In fact, modifiable risk factors such as the hypertension linked to AD promote the degeneration of the vascular system and the reduction of its regenerative capacity [72].

## 7. The Renin-Angiotensin System (RAS) in Alzheimer's Disease

Importantly, a body of accumulating evidence has suggested an association between hypertension and an increased risk of developing AD [73]. The renin-angiotensin system (RAS) is involved in pathological mechanisms of target organ damage as well as the induction of hypertension. The RAS is a hormonal cascade that functions in the homeostatic control of arterial pressure, tissue perfusion, extracellular volume, and cerebral blood flow regulation. Beyond its antihypertensive effects, blockade of the RAS has been expected to prevent cardiovascular and cerebrovascular diseases. Currently, three classes of RAS-targeting drugs are licensed for treatment of peripheral hypertension-angiotensin-converting enzyme inhibitors (ACEIs), angiotensin II receptor blockers (ARBs), and direct renin inhibitors (DRIs). All of these are generally well tolerated and have been shown to offer varying degrees of protection for aspects of cognition and dementia, thus making them an attractive therapeutic option for AD. Angiotensin II, a major player in RAS mainly via the angiotensin type 1 (AT1) receptor, plays an important role in the pathophysiology of tissue dysfunction [74]. The effects of brain angiotensin II depend on AT1 receptor stimulation and its elevated activity is associated with hypertension, heart failure, brain ischemia, abnormal stress responses, blood-brain barrier breakdown, and inflammation [75]. Previous reports indicate the possibility that treatment with antihypertensive agents helps to avoid the impairment of the patient's quality of life, including cognitive performance [76]. Therefore, RAS blockade by ARBs and ACEIs, which are widely used as antihypertensive drugs, is expected to prevent cerebral neurodegenerative disorders.

**Figure 2:**
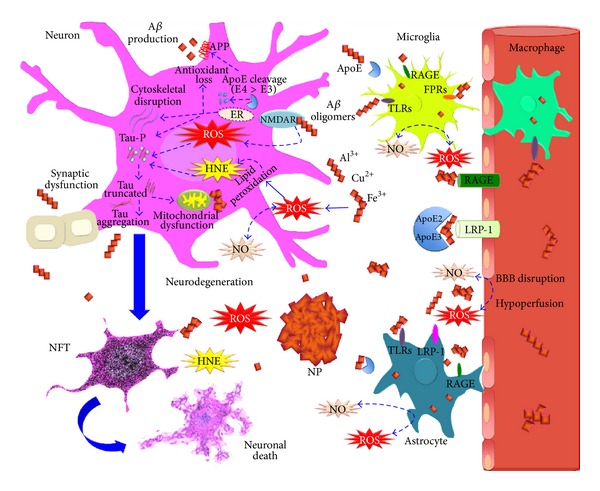
Oxidative stress in Alzheimer's disease. High levels of oxidative stress have been linked with neurodegeneration in AD. It has been thought that amyloid-beta (A*β*) aggregates could be the major inducers of oxidative stress. A*β* overactivates glutamate receptor (NMDAR), promoting a Ca^2+^ influx and the increased generation of reactive oxygen and nitrosative species (ROS and RNS) in mitochondria and endoplasmic reticulum (ER). ROS and RNS may accelerate tau hyperphosphorylation and truncation, which leads to neurofibrillary tangles (NFT) and contributes to neuronal death. Moreover, tau aggregates promote mitochondrial dysfunction and favor oxidative stress generation. In the presence of trace amounts of Fe^3+^, Cu^2+^, and Al^3+^, A*β* aggregates induce membrane lipid peroxidation and the production of 4-hydroxynonenal (HNE), which causes membrane depolarization, Ca^2+^ influx, and tau aggregation. A*β* aggregates also activate microglial cells and astrocytes through Toll-like receptors (TLRs), low density lipoprotein receptor-related protein 1 (LRP-1), the receptor for advanced glycation endproducts (RAGE), and the N-formyl peptide receptors (FPRs), promoting A*β* phagocytosis. At the same time, they could raise ROS and RNS extracellular levels, possibly favoring A*β* aggregation. Apolipoprotein E (ApoE) participates in A*β* clearance from the CNS to the microvasculature through LRP-1 and RAGE, but this effect is attributed mainly to the ApoE2 and ApoE3 isoforms. ApoE2 > ApoE3 have also been reported as having an antioxidant role. In contrast, ApoE4 isoform in AD pathology is linked to the risk of losing the antioxidant system, cytoskeletal dysfunction, tau phosphorylation, and increased APP processing and A*β* production. Despite the fact that not all patients with AD are carriers of ApoE4 isoform, it has been suggested that ApoE undergoes conformational changes that promote those toxic effects. Finally, the chronic increase of oxidative adducts in CNS favors the protein aggregation and mitochondrial and synaptic dysfunction that leads to neuronal death. In addition, the oxidative damage and A*β* aggregates promote a blood brain barrier (BBB) disruption that alters the blood perfusion in the brain. Chronic hypoperfusion impairs endothelium vascular regeneration, a predictor of metabolic syndrome.

Recent studies [77, 78] have demonstrated that angiotensin II increases the production of ROS in cerebral microvessels via gp91phox (nox2), a subunit of NADPH oxidase. Moreover, it has also been recently demonstrated that the slow infusion of the pressor angiotensin II causes the attenuation of the increase in cerebral blood flow induced by both neural activity and endothelium-dependent vasodilators, without the elevation of mean arterial pressure (MAP) [79]. This effect of angiotensin II reduces blood supply and contributes to the patient's increased susceptibility to dementia. The possible beneficial effect of RAS blockade on cognitive function is also being highlighted in the clinical field [76]. It has been shown that male subjects treated with ARBs exhibited a significant reduction in the incidence and progression of AD and dementia compared with those treated with ACEIs and other cardiovascular drugs [80]. Interestingly, in other reports, patients diagnosed with dementia had fewer prescriptions for ARBs and ACEIs and inverse associations with AD were stronger for ARBs compared with ACEIs [81]. Another study by Takeda et al. [82] demonstrated that pretreatment with a low dose of the ARB, olmesartan, completely prevented *β*-amyloid-induced vascular dysregulation and partially attenuated the impairment of hippocampal synaptic plasticity via a decrease in oxidative stress in brain microvessels. Therefore, the blockade of the RAS has been expected to help prevent cardiovascular and cerebrovascular diseases above and beyond its antihypertensive effects. In spite of the well-characterized role of angiotensin (Ang) II receptor blockers (ARBs) in preventing the onset and recurrence of stroke, the clinical evidence for the effect of ARBs on dementia has not been definitive [83]. However, preliminary experiments raise the possibility that treatment using ARBs may prevent ischemic brain damage and cognitive impairment. Moreover, recent reports have shown that some ARBs prevent amyloid-beta deposition in the brain and attenuate cognitive impairment in models of AD. Furthermore, recent cohort studies indicate that lower incidence of AD is observed in elderly individuals treated with ARBs. These results indicate a beneficial role for ARBs in the treatment of the cognitive impairment associated with vascular disease, AD, metabolic syndrome, and other neurodegenerative diseases (Figure 2). Here, we review the effects of ARBs on the brain with a focus on both dementia and future therapeutic approaches for elderly people suffering from disabilities.

In conclusion, many efforts are made to find the mechanisms involved in the pathobiology of AD, with many new therapeutic strategies being focused on the cerebral endothelium. Oxidative stress is an ideal target for drug therapy as it is present in a diverse range of conditions. Takeda el al. [82] showed that olmesartan, an ARB, improved neurovascular dysfunction and decreased ROS production in AD-model transgenic mice. There is now considerable evidence to indicate the importance of cerebrovascular dysfunction in the pathogenesis of AD [77, 84, 85]. ARBs are well tolerated, have beneficial cardiovascular metabolic profiles, and are commonly used for the treatment of hypertension. Recently ARBs have also been used to ameliorate neurodegenerative disorders and to increase the quality of life of AD patients. Interestingly, several studies *in vitro* have shown that ACE inhibitors can reduce cognitive decline. Dong et al. [86] showed that perindopril, a brain penetrating ACEI, protected against cognitive impairment and brain injury in AD mouse model, although this finding was controversial in light of other studies where ACEI was shown to have no beneficial effects on cognitive decline or AD [87, 88]. Interestingly, it has been shown that the use of the ACEIs in older adults with AD is associated with a slower rate of cognitive decline independent of hypertension. Qiu et al. proved that ACEIs were associated with a reduced risk of AD in the absence of ApoE4 but had no such effect in those carrying the ApoE4 allele [89]. Csiszar et al. assessed changes in hippocampal mRNA expression of genes involved in amyloid precursor protein (APP) in young and older angiotensin-induced hypertension mice they reported that hypertension in aging did not increase the expression of APP but demonstrated an association between aged hypertensive mice and spatial memory impairments [90]. Therefore, blockade of the RAS has been expected to prevent cardiovascular and cerebrovascular diseases beyond its antihypertensive effects. In spite of the well-characterized role of ARBs and ACEIs in preventing the damage of the cognitive function, the clinical evidence for an effect of these drugs on dementia has not been definitive.

Taken together, all these studies suggest that lowering the effect of angiotensin II could be a novel therapeutic target in the treatment of AD and dementia. It should be noted that the regulating effects of ARBs and ACEI on cognitive function and AD should be confirmed with carefully designed clinical trials.

### 7.1. Metabolic Syndrome and AD

Metabolic syndrome is cluster of risk factors including insulin resistance, dyslipidemias, abdominal obesity and arterial hypertension. MS itself, as well as obesity, and insulin resistance, is a risk factor for dementia, especially AD [87]. The pathogenesis of MS is complex; however, one remarkable characteristic is the enhanced production of reactive oxygen species. The high levels of circulating lipids increase the lipoperoxidation of lipids, which diminish the antioxidant systems such as superoxide dismutase and catalase and cause, as a consequence, the high levels of oxidative metabolism which affects cell structure, causing neuronal damage. This has been evident in the brains of AD patients, which show augmented levels of oxidized proteins, elevated levels of protein nitrosylation and carbonylation, lipid peroxidation, and RNA and DNA oxidation, as well as sugar modifications and the presence of ROS [88, 91].

Insulin resistance (IR) is the main characteristic of MS. IR is brought about by the incapacity of cells to respond to hormonal stimulus, especially in skeletal and cardiac muscle, adipose tissue, and the CNS itself. In the brain, it is believed that IR has an important role in APP and tau protein metabolism, since it increases A*β* accumulation and buildup of NP (Figure 2) [87].

IR causes a decrease in glucose utilization in brain tissues, which is the principal source of energy production. It is well known that the energy metabolism of neurons is deteriorated in brains affected by AD and that this energy deficit can be attributed to changes in insulin-dependent glucose uptake and damage to the different proteins that participate in the glucose metabolism [92]. Positron emission tomography performed in AD patients shows a progressive reduction of glucose metabolism as well as diminished blood flow in the parietal and temporal lobes, which correlates with the severity of dementia in these patients [93]. It has been proposed that IR may be conducive to lipid toxicity and subsequent enhanced lipoperoxidation and increased ROS production [94].

In addition, obesity contributes to AD development through the excessive production of inflammatory cytokines by adipose tissue; leptin, tumor necrosis factor (TNF*α*), and adiponectin, as well as the interleukins IL*β*, and IL6 are among them. The postulated participation of leptin in the development of neurodegeneration is controversial. It has been demonstrated that leptin is neuroprotective cytokine, since it inhibits formation of neuritic plaques. Thus, it is hard to explain why neurodegeneration is increased in patients suffering from MS, since the condition usually causes hyperleptinemia. This may be partially explained by resistance to this hormone in peripheral tissues, causing augmented secretion of this peptide as well as diminished leptin transport across the BBB [95]. Similarly, it has been demonstrated that TNF*α* is overexpressed in the adipose tissue of obese insulin-resistant rodents and humans as well as in the brains of AD patients and adults with mild cognitive impairment. All of this strongly suggests that increased TNF*α*-associated with obesity, insulin resistance, and hyperinsulinemia can cause an elevation in the cerebral accretion of A*β* or increased neurodegenerative processes [96].

## 8. Conclusion

Impairment of energy metabolism, insulin resistance, and inflammation are three of the most important prompters of ROS production; all three may accelerate the neurodegenerative processes leading to the development of AD. Although the exact mechanisms underlying the connection between MS and AD remain uncertain, it is known that, together, amyloid deposition, vascular damage, and impairment of energy metabolism and insulin resistance are physiological conditions that favor the development of AD. Despite this hypothesis, it is unknown whether oxidative stress and metabolic syndrome are causes or consequences of amyloid toxicity.

However, it is clear that oxidative stress plays an important role in the development of AD and other neurodegenerative diseases, even if or if not MS is present. In this sense, all these insights may be taken in account to develop new therapies in treatment of MS and AD, focused on targeting sources of ROS production and antioxidant molecules. Moreover, since energy metabolism is crucially affected during MS, protection of fuel producing machinery in the cell, such as mitochondria, and energy transfer systems, such as creatine kinase, could stop the extent of the damage caused by metabolism derangement.

## References

[1] Moreira PI, Nunomura A, Nakamura M (2008). Nucleic acid oxidation in Alzheimer disease. *Free Radical Biology and Medicine*.

[2] Ashe KH, Zahs KR (2010). Probing the biology of Alzheimer’s disease in mice. *Neuron*.

[3] Zahs KR, Ashe KH (2013). beta-Amyloid oligomers in aging and Alzheimer's disease. *Frontiers in Aging Neuroscience*.

[4] Sakono M, Zako T (2010). Amyloid oligomers: formation and toxicity of A*β* oligomers. *The FEBS Journal*.

[5] Walsh DM, Selkoe DJ (2004). Oligomers in the brain: the emerging role of soluble protein aggregates in neurodegeneration. *Protein and Peptide Letters*.

[6] Puzzo D, Privitera L, Leznik E (2008). Picomolar amyloid-*β* positively modulates synaptic plasticity and memory in hippocampus. *Journal of Neuroscience*.

[7] Puzzo D, Privitera L, Palmeri A (2012). Hormetic effect of amyloid-beta peptide in synaptic plasticity and memory. *Neurobiology of Aging*.

[8] Koffie RM, Hyman BT, Spires-Jones TL (2011). Alzheimer’s disease: synapses gone cold. *Molecular Neurodegeneration*.

[9] Reddy PH, Beal MF (2008). Amyloid beta, mitochondrial dysfunction and synaptic damage: implications for cognitive decline in aging and Alzheimer’s disease. *Trends in Molecular Medicine*.

[10] Butterfield DA, Boyd-Kimball D (2005). The critical role of methionine 35 in Alzheimer’s amyloid *β*-peptide (1-42)-induced oxidative stress and neurotoxicity. *Biochimica et Biophysica Acta*.

[11] Butterfield DA, Bush AI (2004). Alzheimer’s amyloid *β*-peptide (1-42): involvement of methionine residue 35 in the oxidative stress and neurotoxicity properties of this peptide. *Neurobiology of Aging*.

[12] Butterfield DA, Kanski J (2002). Methionine residue 35 is critical for the oxidative stress and neurotoxic properties of Alzheimer’s amyloid *β*-peptide 1-42. *Peptides*.

[13] Butterfield DA, Drake J, Pocernich C, Castegna A (2001). Evidence of oxidative damage in Alzheimer’s disease brain: central role for amyloid *β*-peptide. *Trends in Molecular Medicine*.

[14] Weingarten MD, Lockwood AH, Hwo SY, Kirschner MW (1975). A protein factor essential for microtubule assembly. *Proceedings of the National Academy of Sciences of the United States of America*.

[15] Goedert M, Spillantini MG, Jakes R, Rutherford D, Crowther RA (1989). Multiple isoforms of human microtubule-associated protein tau: sequences and localization in neurofibrillary tangles of Alzheimer’s disease. *Neuron*.

[16] Brandt R, Léger J, Lee G (1995). Interaction of tau with the neural plasma membrane mediated by tau’s amino-terminal projection domain. *Journal of Cell Biology*.

[17] Goode BL, Denis PE, Panda D (1997). Functional interactions between the proline-rich and repeat regions of tau enhance microtubule binding and assembly. *Molecular Biology of the Cell*.

[18] Mukrasch MD, Biernat J, von Bergen M, Griesinger C, Mandelkow E, Zweckstetter M (2005). Sites of tau important for aggregation populate *β*-structure and bind to microtubules and polyanions. *Journal of Biological Chemistry*.

[19] Braak H, Braak E (1991). Neuropathological stageing of Alzheimer-related changes. *Acta Neuropathologica*.

[20] Lasagna-Reeves CA, Castillo-Carranza DL, Sengupta U (2012). Identification of oligomers at early stages of tau aggregation in Alzheimer's disease. *The FASEB Journal*.

[21] Patterson KR, Remmers C, Fu Y (2011). Characterization of prefibrillar tau oligomers in vitro and in Alzheimer disease. *Journal of Biological Chemistry*.

[22] Rapoport SI (2003). Coupled reductions in brain oxidative phosphorylation and synaptic function can be quantified and staged in the course of Alzheimer disease. *Neurotoxicity Research*.

[23] Lasagna-Reeves CA, Castillo-Carranza DL, Sengupta U, Clos AL, Jackson GR, Kayed R (2011). Tau oligomers impair memory and induce synaptic and mitochondrial dysfunction in wild-type mice. *Molecular Neurodegeneration*.

[24] Martin L, Latypova X, Terro F (2011). Post-translational modifications of tau protein: implications for Alzheimer’s disease. *Neurochemistry International*.

[25] Quintanilla RA, Matthews-Roberson TA, Dolan PJ, Johnsion GVW (2009). Caspase-cleaved tau expression induces mitochondrial dysfunction in immortalized cortical neurons: implications for the pathogenesis of Alzheimer disease. *Journal of Biological Chemistry*.

[26] Lasagna-Reeves CA, Castillo-Carranza DL, Guerrero-Muñoz MJ, Jackson GR, Kayed R (2010). Preparation and characterization of neurotoxic tau oligomers. *Biochemistry*.

[27] Ferrari A, Hoerndli F, Baechi T, Nitsch RM, Götz J (2003). *β*-amyloid induces paired helical filament-like tau filaments in tissue culture. *Journal of Biological Chemistry*.

[28] Good PF, Perl DP, Bierer LM, Schmeidler J (1992). Selective accumulation of aluminum and iron in the neurofibrillary tangles of Alzheimer’s disease: a laser microprobe (LAMMA) study. *Annals of Neurology*.

[29] Smith MA, Harris PLR, Sayre LM, Perry G (1997). Iron accumulation in Alzheimer disease is a source of redox-generated free radicals. *Proceedings of the National Academy of Sciences of the United States of America*.

[30] Resende R, Moreira PI, Proença T (2008). Brain oxidative stress in a triple-transgenic mouse model of Alzheimer disease. *Free Radical Biology and Medicine*.

[31] Su B, Wang X, Lee H-G (2010). Chronic oxidative stress causes increased tau phosphorylation in M17 neuroblastoma cells. *Neuroscience Letters*.

[32] Liu Q, Smith MA, Avilá J (2005). Alzheimer-specific epitopes of tau represent lipid peroxidation-induced conformations. *Free Radical Biology and Medicine*.

[33] Zhang Y-J, Xu Y-F, Liu Y-H (2006). Peroxynitrite induces Alzheimer-like tau modifications and accumulation in rat brain and its underlying mechanisms. *The FASEB Journal*.

[34] Mahley RW (1988). Apolipoprotein E: cholesterol transport protein with expanding role in cell biology. *Science*.

[35] Hatters DM, Peters-Libeu CA, Weisgraber KH (2006). Apolipoprotein E structure: insights into function. *Trends in Biochemical Sciences*.

[36] Corder EH, Saunders AM, Strittmatter WJ (1993). Gene dose of apolipoprotein E type 4 allele and the risk of Alzheimer’s disease in late onset families. *Science*.

[37] Munson GW, Roher AE, Kuo Y-M (2000). SDS-stable complex formation between native apolipoprotein E3 and *β*-amyloid peptides. *Biochemistry*.

[38] Holtzman DM (2001). Role of apoE/A*β* interactions in the pathogenesis of Alzheimer’s disease and cerebral amyloid angiopathy. *Journal of Molecular Neuroscience*.

[39] Holtzman DM, Bales KR, Tenkova T (2000). Apolipoprotein E isoform-dependent amyloid deposition and neuritic degeneration in a mouse model of Alzheimer’s disease. *Proceedings of the National Academy of Sciences of the United States of America*.

[40] Ye S, Huang Y, Müllendorff K (2005). Apolipoprotein (apo) E4 enhances amyloid *β* peptide production in cultured neuronal cells: ApoE structure as a potential therapeutic target. *Proceedings of the National Academy of Sciences of the United States of America*.

[41] Hayek T, Oiknine J, Brook JG, Aviram M (1994). Increased plasma and lipoprotein lipid peroxidation in apo E-deficient mice. *Biochemical and Biophysical Research Communications*.

[42] Shea TB, Rogers E, Ashline D, Ortiz D, Sheu M-S (2002). Apolipoprotein E deficiency promotes increased oxidative stress and compensatory increases in antioxidants in brain tissue. *Free Radical Biology and Medicine*.

[43] Miyata M, Smith JD (1996). Apolipoprotein E allele-specific antioxidant activity and effects on cytotoxicity by oxidative insults and *β*-amyloid peptides. *Nature Genetics*.

[44] Kharrazi H, Vaisi-Raygani A, Rahimi Z, Tavilani H, Aminian M, Pourmotabbed T (2008). Association between enzymatic and non-enzymatic antioxidant defense mechanism with apolipoprotein E genotypes in Alzheimer disease. *Clinical Biochemistry*.

[45] Lauderback CM, Kanski J, Hackett JM, Maeda N, Kindy MS, Butterfield DA (2002). Apolipoprotein E modulates Alzheimer’s A*β*(1-42)-induced oxidative damage to synaptosomes in an allele-specific manner. *Brain Research*.

[46] Ramassamy C, Averill D, Beffert U (1999). Oxidative damage and protection by antioxidants in the frontal cortex of Alzheimer’s disease is related to the apolipoprotein E genotype. *Free Radical Biology and Medicine*.

[47] Tchantchou F, Graves M, Rogers E, Ortiz D, Shea TB (2005). N-acteyl cysteine alleviates oxidative damage to central nervous system of ApoE-deficient mice following folate and vitamin E-deficiency. *Journal of Alzheimer’s Disease*.

[48] Huebbe P, Jofre-Monseny L, Boesch-Saadatmandi C, Minihane A-M, Rimbach G (2007). Effect of apoE genotype and vitamin E on biomarkers of oxidative stress in cultured neuronal cells and the brain of targeted replacement mice. *Journal of Physiology and Pharmacology*.

[49] Saxton WM, Hollenbeck PJ (2012). The axonal transport of mitochondria. *Journal of Cell Science*.

[50] Li Z, Okamoto K-I, Hayashi Y, Sheng M (2004). The importance of dendritic mitochondria in the morphogenesis and plasticity of spines and synapses. *Cell*.

[51] Bernard G, Bellance N, James D (2007). Mitochondrial bioenergetics and structural network organization. *Journal of Cell Science*.

[52] Du H, Guo L, Yan S, Sosunov AA, McKhann GM, Yan SS (2010). Early deficits in synaptic mitochondria in an Alzheimer’s disease mouse model. *Proceedings of the National Academy of Sciences of the United States of America*.

[53] Bobba A, Amadoro G, Valenti D, Corsetti V, Lassandro R, Atlante A (2013). Mitochondrial respiratory chain Complexes I and IV are impaired by beta-amyloid via direct interaction and through Complex I-dependent ROS production, respectively. *Mitochondrion*.

[54] Calkins MJ, Manczak M, Mao P, Shirendeb U, Reddy PH (2011). Impaired mitochondrial biogenesis, defective axonal transport of mitochondria, abnormal mitochondrial dynamics and synaptic degeneration in a mouse model of Alzheimer’s disease. *Human Molecular Genetics*.

[55] Tretter L, Sipos I, Adam-Vizi V (2004). Initiation of neuronal damage by complex I deficiency and oxidative stress in Parkinson’s disease. *Neurochemical Research*.

[56] Grijalba MT, Vercesi AE, Schreier S (1999). Ca^2+^-induced increased lipid packing and domain formation in submitochondrial particles. A possible early step in the mechanism of Ca^2+^- stimulated generation of reactive oxygen species by the respiratory chain. *Biochemistry*.

[57] Wang X, Su B, Lee H-G (2009). Impaired balance of mitochondrial fission and fusion in Alzheimer’s disease. *Journal of Neuroscience*.

[58] Manczak M, Calkins MJ, Reddy PH (2011). Impaired mitochondrial dynamics and abnormal interaction of amyloid beta with mitochondrial protein Drp1 in neurons from patients with Alzheimer’s disease: implications for neuronal damage. *Human Molecular Genetics*.

[59] Mjaatvedt AE, Wong-Riley MTT (1988). Relationship between synaptogenesis and cytochrome oxidase activity in Purkinje cells of the developing rat cerebellum. *Journal of Comparative Neurology*.

[60] Jendrach M, Mai S, Pohl S, Vöth M, Bereiter-Hahn J (2008). Short- and long-term alterations of mitochondrial morphology, dynamics and mtDNA after transient oxidative stress. *Mitochondrion*.

[61] Jost CR, van der Zee CEEM, In ’t Zandt HJA (2002). Creatine kinase B-driven energy transfer in the brain is important for habituation and spatial learning behaviour, mossy fibre field size and determination of seizure susceptibility. *European Journal of Neuroscience*.

[62] Streijger F, Oerlemans F, Ellenbroek BA, Jost CR, Wieringa B, van der Zee CEEM (2005). Structural and behavioural consequences of double deficiency for creatine kinases BCK and UbCKmit. *Behavioural Brain Research*.

[63] Bürklen TS, Schlattner U, Homayouni R (2006). The creatine kinase/creatine connection to alzheimer’s disease: CK-inactivation, APP-CK complexes and focal creatine deposits. *Journal of Biomedicine and Biotechnology*.

[64] Iadecola C (2004). Neurovascular regulation in the normal brain and in Alzheimer’s disease. *Nature Reviews Neuroscience*.

[65] Park L, Anrather J, Zhou P (2005). NADPH oxidase-derived reactive oxygen species mediate the cerebrovascular dysfunction induced by the amyloid *β* peptide. *Journal of Neuroscience*.

[66] Park L, Zhou P, Pitstick R (2008). Nox2-derived radicals contribute to neurovascular and behavioral dysfunction in mice overexpressing the amyloid precursor protein. *Proceedings of the National Academy of Sciences of the United States of America*.

[67] Leszek J, Sochocka M, Gasiorowski K (2012). Vascular factors and epigenetic modifications in the pathogenesis of Alzheimer's disease. *Journal of the Neurological Sciences*.

[68] Luscher TF, Noll G (1995). The pathogenesis of cardiovascular disease: role of the endothelium as a target and mediator. *Atherosclerosis*.

[69] Kim HA, Miller AA, Drummond GR (2012). Vascular cognitive impairment and Alzheimer's disease: role of cerebral hypoperfusion and oxidative stress. *Naunyn-Schmiedeberg's Archives of Pharmacology*.

[70] Sochocka M, Koutsouraki ES, Gasiorowski K, Leszek J (2013). Vascular oxidative stress and mitochondrial failure in the pathobiology of Alzheimer's disease: new approach to therapy. *CNS and Neurological Disorders*.

[71] Aliev G, Palacios HH, Walrafen B, Lipsitt AE, Obrenovich ME, Morales L (2009). Brain mitochondria as a primary target in the development of treatment strategies for Alzheimer disease. *International Journal of Biochemistry and Cell Biology*.

[72] Akinyemi RO, Mukaetova-Ladinska EB, Attems J, Ihara M, Kalaria RN (2013). Vascular risk factors and neurodegeneration in ageing related dementias: Alzheimer's disease and vascular dementia. *Current Alzheimer Research*.

[73] Nelson L, Tabet N, Richardson C, Gard P (2013). Antihypertensives, angiotensin, glucose and Alzheimer's disease. *Expert Review of Neurotherapeutics*.

[74] Schmieder RE, Hilgers KF, Schlaich MP, Schmidt BM (2007). Renin-angiotensin system and cardiovascular risk. *The Lancet*.

[75] Saavedra JM (2012). Angiotensin II AT(1) receptor blockers as treatments for inflammatory brain disorders. *Clinical Science*.

[76] Mogi M, Iwanami J, Horiuchi M (2012). Roles of brain angiotensin II in cognitive function and dementia. *International Journal of Hypertension*.

[77] Girouard H, Park L, Anrather J, Zhou P, Iadecola C (2006). Angiotensin II attenuates endothelium-dependent responses in the cerebral microcirculation through nox-2-derived radicals. *Arteriosclerosis, Thrombosis, and Vascular Biology*.

[78] Kazama K, Anrather J, Zhou P (2004). Angiotensin II impairs neurovascular coupling in neocortex through NADPH oxidase-derived radicals. *Circulation Research*.

[79] Capone C, Faraco G, Park L, Cao X, Davisson RL, Iadecola C (2011). The cerebrovascular dysfunction induced by slow pressor doses of angiotensin II precedes the development of hypertension. *The American Journal of Physiology*.

[80] Li NC, Lee A, Whitmer RA (2010). Use of angiotensin receptor blockers and risk of dementia in a predominantly male population: prospective cohort analysis. *BMJ*.

[81] Davies NM, Kehoe PG, Ben-Shlomo Y, Martin RM (2011). Associations of anti-hypertensive treatments with Alzheimer’s disease, vascular dementia, and other dementias. *Journal of Alzheimer’s Disease*.

[82] Takeda S, Sato N, Takeuchi D (2009). Angiotensin receptor blocker prevented *β*-amyloid-induced cognitive impairment associated with recovery of neurovascular coupling. *Hypertension*.

[83] Mogi M, Horiuchi M (2009). Effects of angiotensin II receptor blockers on dementia. *Hypertension Research*.

[84] de La Torre JC (2004). Alzheimer’s disease is a vasocognopathy: a new term to describe its nature. *Neurological Research*.

[85] Zlokovic BV (2005). Neurovascular mechanisms of Alzheimer’s neurodegeneration. *Trends in Neurosciences*.

[86] Dong YF, Kataoka K, Tokutomi Y (2011). Perindopril, a centrally active angiotensin-converting enzyme inhibitor, prevents cognitive impairment in mouse models of Alzheimer’s disease. *The FASEB Journal*.

[87] Milionis HJ, Florentin M, Giannopoulos S (2008). Metabolic syndrome and alzheimer’s disease: a link to a vascular hypothesis?. *CNS Spectrums*.

[88] Drake J, Link CD, Butterfield DA (2003). Oxidative stress precedes fibrillar deposition of Alzheimer’s disease amyloid *β*-peptide (1-42) in a transgenic Caenorhabditis elegans model. *Neurobiology of Aging*.

[89] Qiu WQ, Mwamburi M, Besser LM (2013). Angiotensin converting enzyme inhibitors and the reduced risk of Alzheimer's disease in the absence of apolipoprotein E4 allele. *Journal of Alzheimer's Disease*.

[90] Csiszar A, Tucsek Z, Toth P (2013). Synergistic effects of hypertension and aging on cognitive function and hippocampal expression of genes involved in beta-amyloid generation and Alzheimer's disease. *The American Journal of Physiology. Heart and Circulatory Physiology*.

[91] LaFontaine MA, Mattson MP, Butterfield DA (2002). Oxidative stress in synaptosomal proteins from mutant presenilin-1 knock-in mice: implications for familial Alzheimer’s disease. *Neurochemical Research*.

[92] Bosco D, Fava A, Plastino M, Montalcini T, Pujia A (2011). Possible implications of insulin resistance and glucose metabolism in Alzheimer’s disease pathogenesis. *Journal of Cellular and Molecular Medicine*.

[93] Friedland RP, Budinger TF, Ganz E (1983). Regional cerebral metabolic alterations in dementia of the Alzheimer type: positron emission tomography with [^18^F]fluorodeoxyglucose. *Journal of Computer Assisted Tomography*.

[94] Erdös B, Snipes JA, Miller AW, Busija DW (2004). Cerebrovascular dysfunction in zucker obese rats is mediated by oxidative stress and protein kinase C. *Diabetes*.

[95] Pasinetti GM, Eberstein JA (2008). Metabolic syndrome and the role of dietary lifestyles in Alzheimer’s disease. *Journal of Neurochemistry*.

[96] Craft S (2009). The role of metabolic disorders in Alzheimer disease and vascular dementia: two roads converged. *Archives of Neurology*.

